# Effect of Dietary Supplementation with Organic Silicon on the Growth Performance, Blood Biochemistry, Digestive Enzymes, Morphohistology, Intestinal Microbiota and Stress Resistance in Juvenile Hybrid Tilapia (*Oreochromis mossambicus* × *Oreochromis niloticus*)

**DOI:** 10.3390/biology13070531

**Published:** 2024-07-17

**Authors:** Yuniel Méndez-Martínez, Helen A. Valensuela-Barros, Yanis Cruz-Quintana, Aroldo Botello-León, Roberto D. Muñoz-Mestanza, Grace L. Orellana-Castro, Carlos Angulo

**Affiliations:** 1 Experimental Aquaculture Laboratory, Facultad de Ciencias Pecuarias y Biológicas, Universidad Técnica Estatal de Quevedo (UTEQ), Av. Quito Km. 1 1/2 via a Santo Domingo de los Tsáchilas, Quevedo 120301, Los Ríos, Ecuador; hvalenzuelab@uteq.edu.ec (H.A.V.-B.); rmunozm5@uteq.edu.ec (R.D.M.-M.);; 2 Grupo de Investigación en Sanidad Acuícola, Inocuidad y Salud Ambiental (SAISA), Departamento de Acuicultura, Pesca y Recursos Naturales Renovables, Facultad de Acuicultura y Ciencias del Mar, Universidad Técnica de Manabí (UTM), c/Gonzalo Loor Velasco s/n, Bahía de Caráquez 130104, Manabí, Ecuador; yanis.cruz@utm.edu.ec; 3 Aquaculture Laboratory, Facultad de Ciencias Agropecuarias, Universidad Técnica Luis Vargas Torres de Esmeraldas (UTLVTE), Km 18 via Aeropuerto, San Mateo 080150, Esmeraldas, Ecuador; aroldo.botello@utelvt.edu.ec; 4 Immunology & Vaccinology Group, Centro de Investigaciones Biológicas del Noroeste (CIBNOR), Av. Instituto Politecnico Nacional #195, Playa Palo de Santa Rita Sur, La Paz 23096, Baja California Sur, Mexico

**Keywords:** feeding efficiency, digestive physiology, hypoxia stress, mineral supplement, functional food

## Abstract

**Simple Summary:**

This study evaluated the effect of dietary supplementation with organic silicon on the growth performance, blood biochemistry, digestive enzymes, morphohistology, intestinal microbiota and stress resistance in juvenile hybrid Tilapia (*Oreochromis mossambicus × Oreochromis niloticus*). It is concluded that organic silicon had no negative effects on the physiology and metabolism of the fish. The results showed that up to 50 mg·kg^−1^ of organic silicon can be applied to the diet of red Tilapia, improving digestive, metabolic and growth performance. The interactions between zootechnical performance, physiological digestive and metabolic capacity in hybrid red Tilapia still deserve further exploration.

**Abstract:**

In recent decades, interest has been aroused worldwide in the use of silicon in nutrition; however, information on its effect on nutrition and metabolism of fish is limited. The objective of the research was to evaluate the effect of dietary supplementation with organic silicon on the growth performance, blood biochemistry, digestive enzymes, morphohistology and intestinal microbiota and stress resistance in hybrid Tilapia (*Oreochromis mossambicus × Oreochromis niloticus*). Methodologically, six levels of organic silicon (DOS) [control (0), 10, 20, 30, 40 and 50 mg·kg^−1^] were used to feed juvenile fish (initial weight 7.51 ± 0.25 g) grown for eight weeks in 18 aquariums (15 fish/aquarium). The results indicated that growth performance showed differences (*p* < 0.05) for specific growth rate, feed conversion and survival. Triglycerides, cholesterol and glucose, transaminases and digestive enzymes were significantly influenced by DOS levels. The histological study confirmed that the administered diets did not cause damage and induced significant morphological changes in the proximal intestine. The 16S rRNA gene sequencing analysis of the gut microbiota showed a high diversity and richness of OTU/Chao-1, with Fusobacteria, Proteobacteria, Bacteroidetes and Acidobacteria predominating in the DOS treatments compared to the control (*p* < 0.05). Induction of hypoxia stress after the feeding period showed a significant relative survival rate of 83.33% in fish fed 50 mg·kg^−1^. It is concluded that the DOS treatments performed better than the control treatment in most of the variables analysed. DOS had no negative effects on the fish. The results showed that up to 50 mg·kg^−1^ DOS improved digestive, metabolic and growth performance in hybrid Tilapia.

## 1. Introduction

On a global scale, the farming of aquatic animal species in inland environments in recent years represents approximately 62.2% of the total production of the aquaculture industry [1]. Among the cultivated species is red Tilapia, also known as “mojarra roja”, the result of hybridisation between several species [2,3]. Its origin is in southern Central Africa [4,5], and its worldwide distribution has increased due to its commercial and social value, high protein content, diversity of unsaturated fatty acids and organoleptic conditions [6]. The harvest is expected to reach 7.3 million tonnes by 2030, with China, Indonesia and Egypt being the main producers [7]. Tilapia farming is viable in both freshwater and saltwater, making it an alternative food source for developing countries, such as those in Latin America [8,9]. The cultivation of red Tilapia was introduced in 1993 in the country of Ecuador, and currently it is the second most important species in aquaculture after white shrimp (*Penaeus vannamei*).

However, successful production depends to a large extent on the efficient management of each aspect of production. Among these, feed on a global scale could be considered the most crucial factor, as it can represent 40 to 60% of total production costs [10]; inadequate feed management or nutritional imbalance can lead to the proliferation of opportunistic pathogens, causing extreme numbers of those pathogens, stress and low survival rates that affect productive performance, mainly in intensive systems where there are high densities of Tilapia in culture. As an alternative, various producers use chemicals and drugs such as antibiotics [11]. However, these products have a limited spectrum of action, and their prolonged and uncontrolled use induces the development of pathogenic microorganisms resistant to antibiotics and causes negative effects on the environment.

However, various ecosystem-friendly solutions are being explored to improve and increase productivity and feed efficiency in Tilapia farming [12]. Animal experiments have shown that simple practices, such as certain dietary changes, generate variations in digestive physiology, metabolism, the microbial profile of the intestinal tract, improved resistance to certain parasitic diseases and greater tolerance to stress, contributing to animal health and welfare and the preservation of the environment [13,14]. In this regard, some alternatives, such as prebiotics or biostimulants, contain dietary minerals that participate in various metabolic processes in fish and are considered trace elements [15,16]. New minerals acting as prebiotics, used in aquaculture, improve feed production processes. Among these minerals, which have a broad range of action mechanisms in organisms, silicon stands out [17,18].

Organic silica has been produced since the 1950s, which marked the beginning of silicotherapy. Today, various products containing organic silica are used to improve the health of humans, animals and plants [19,20]. Since the 1930s, research on organic silicon has shown that organic silicon is essential for humans and animals [17,21]. Its deficiency produces alterations and deformations in bone growth, and it can also generate various conditions such as: coronary pathologies, osteoarticular alterations, decreased defences, growth retardation problems, fragile bones, fibrosis and joint flaccidity. In addition, it has been confirmed that individuals who suffer from diseases such as tuberculosis and cancer have a silicon deficiency [19]. Bashar et al. [20] found improvements in digestive physiology, digestibility and growth in Nile tilapia when fed silicon nanoparticles. However, studies on aquatic organisms are very limited. In Ecuador, the use of alternative foods such as organic acids, prebiotics and probiotics is documented [11,12]; however, the use of silicon in the diet of aquatic organisms is not reported.

Silicon is involved in the interaction of fish with their aquatic environment through the ingestion of silicon-containing organisms, the formation of hard structures or participation in physiological processes [22]. The importance and effects of silicon in fish may vary depending on the species and ecosystem in question. Silicon has been reported to enhance leptin response on the activity of the glyco-sensory system and the expression of anorexigenic and orexigenic neuropeptides that participate in the regulation of food intake [23], the digestion and absorption of nutrients, contributing to strengthening of connective tissues and the function of organs such as the stomach and intestine, providing elasticity and strength. It also supports the health of the mucous membranes of the digestive system by participating in the formation of neuropeptides and digestive enzymes [22,24].

Silicon has an important role in strengthening the immune and hematic systems by sending signals that facilitate the formation of antigen–antibody complexes and induce the proliferation of T and B lymphocytes [25].

In addition, it can help reduce susceptibility to disease and pathogens, even in the case of severe overcrowding [26]. It has also been found to have structural activity and is involved in cell growth and bone cartilage biosynthesis [27]. In addition, silica has been shown to be effective in improving water quality [28,29]. In a more synergistic role, it has been reported to reinforce the action of zinc and copper in humans [19]. It has been reported that silica, when combined with other metals such as aluminium, can create hydroxyaluminosilicates AlOSi (OH_3_)^2+^, thus avoiding the toxicity of aluminium [30].

Exhaustive research is required to demonstrate the benefits of dietary supplementation with organic silicon in fish, taking into account species and physiological stage. Reports on the effects of organic silicon on growth, biochemistry, physiology and metabolism of Tilapia are scarce [20].

This study hypothesized that the use of organic silicon may influence fish growth, blood biochemistry, digestive physiology and stress resistance of hybrid Tilapia. Therefore, this study was conducted to evaluate the effect of dietary supplementation with organic silicon on the growth performance, blood biochemistry, digestive enzymes, morphohistology, intestinal microbiota and stress resistance in hybrid Tilapia (*Oreochromis mossambicus × Oreochromis niloticus*).

## 2. Materials and Methods

### 2.1. Study Site

The experiment was carried out in an indoor facility at the Aquaculture Laboratory of the Universidad Técnica Estatal de Quevedo (UTEQ), located in Quevedo, Los Ríos province, Ecuador, whose geographical coordinates are 01 or 06′13″ south latitude and 79 or 29′22″ west longitude with an altitude of 73 masl.

### 2.2. Formulation and Preparation of Experimental Diets

The experimental diet formulations are presented in Table 1 and contain six different levels: control [0%, 10, 20, 30, 40 and 50 mg·kg^−1^] of organic silicon [CH_3_Si(OH)_3_]. The formulation was performed with LINDO software (Formulation Systems software, v-6, Chicago, IL, USA). The macro-ingredients were sieved with a 250 µm mesh. All ingredients were weighed with digital scales. Each diet was prepared by mixing all macro-ingredients in an industrial mixer until a uniform mass was obtained. The micro-ingredients were also mixed individually before being added to the mixture. Soybean oil and fish oil were mixed until a homogeneous mixture was obtained. Water was then added, equivalent to 30% of the weight of the diet ingredients. The feed was passed twice through a meat grinder to form granules or pellets of 2 mm in diameter, which were then dried for 8 h at 45 °C in an airflow oven. All diets were placed in plastic bags and stored at −4 °C.

### 2.3. Chemical Analysis of Diets

The proximate composition of the experimental diets (Table 2) was determined using AOAC methods [31]. All analyses were performed in triplicate. Moisture content was analysed by drying the samples to constant weight at 105 °C. The ashes were incinerated in a muffle for 8 h at 550 °C. Crude protein (N × 6.25) was determined using the Kjeldahl nitrogen combustion method and the ether extract content using the Soxtec System ether extraction method. Fibre was determined according to the Weende method and nitrogen-free extract by difference. Digestible energy was theoretically estimated from conversion factors of 4.25 kcal g^−1^ for animal protein, 3.8 kcal g^−1^ for vegetable protein, 8.0 kcal g^−1^ for lipids, 2.0 kcal g^−1^ for carbohydrates in legumes and 3.0 kcal g^−1^ for carbohydrates in non-legumes, according to Ramanathan et al. [32]. The essential amino acid contribution was calculated according to NRC [33] for red Tilapia (*Oreochromis* spp.).

### 2.4. Experimental Design and Rearing Conditions

Juvenile fish (7.51 ± 0.25 g) were obtained from the Red Tilapia Production and Reproduction Center located in the Puerto Limón parish, province of Santo Domingo, Ecuador, and taken to the UTEQ Aquaculture Laboratory. The fish were acclimatised for seven days before the experiment. Then, the fish were randomly placed in 18 aquariums (*n* = 3 aquariums per treatment), at a density of 15 fish/aquarium with 50 L of water and fed the experimental diets. The bioassay lasted eight weeks.

Water O_2_ was measured with a digital oximeter, water temperature with a mercury thermometer (0 to 50 °C), and pH and NH_4_ with the colorimetric kit (API Saltwater Master, Chalfont, PA, USA). A natural light photoperiod (12 h of light and 12 h of darkness) was used. The physico-chemicals of the water were evaluated daily. The O_2_ of the water was in the range of 5.64 ± 0.68 mg·L^−1^, the temperature at 28.43 ± 0.5 °C, the NH_4_ at 0.05 ± 0.03 mg·L^−1^ and the pH at 7.11 ± 0.09.

All aquariums were siphoned every morning before feeding to remove faeces and excess food, and then the water was replaced. Fish were fed ad libitum twice daily at 09:00 and 17:00. Food intake was determined by feeding to apparent satiation, and food remains, which could be easily identified by their shape, were removed the following morning. Leftover food was quantified by concentrating it on Whatman No.1 filter paper with a vacuum pump and then dried at 50 °C for 18 h in an airflow oven [4]. Daily rations were adjusted each week to minimise the amount of residual feed.

### 2.5. Sample Collection

At the end of the experiment (8 weeks), all fish were fasted for 24 h and then anaesthetised with eugenol (1:10,000) before being weighed and measured. All fish were individually weighed on digital scales (±0.01 g), and their total length was determined with a vernier (±0.001 mm).

Five experimental juvenile red Tilapia were then randomly selected from each aquarium (*n* = 15 per treatment), and blood samples were drawn from the fish by puncturing the caudal artery at the level of the haemal arch using disposable syringes (1 mL). Blood samples were kept at 4 °C for 24 h for blood biochemistry analysis. Then, from the same five juvenile red Tilapia (*n* = 15 per treatment), the midgut and hindgut were dissected for digestive enzyme and gut microbiota analysis, respectively. The midgut was homogenised in a buffer solution (Tris-HCl 30 mM, CaCl_2_ 12.5 mM, pH 7.5), followed by double centrifugation at 14,000 rpm at 4 °C for 15 min, and the supernatants were transferred to fresh tubes and used as a crude enzyme source to analyse the digestive enzyme activity. To analyse the hindgut metagenome, samples were placed in Eppendorf tubes, where 300 mg of tissue was fixed in 500 µL of DNA/RNA Shield^®^ reagent and stored at −20 °C until processing.

Then, three juvenile red Tilapia from each aquarium (*n* = 9 per treatment) were randomly sacrificed with neutral buffered formalin injection to examine the midgut histologically, and samples were immediately preserved by immersion in 10% buffered formalin for 24 h for histological analysis.

At the end of the feeding bioassay, six juvenile red Tilapia from each aquarium (*n* = 18 per treatment) were randomly collected to determine hypoxic stress.

### 2.6. Fish Growth Performance

Mathematical formulae were used to determine specific growth rate, weight gain rate, feed conversion ratio, condition factor and survival rate. These are detailed below:

Weight Gain Rate (%) = [Wx − Wi] × 100

Specific Growth Rate = 100 × [(lnWx − lnWi)/t]

Condition Factor = 100 × [Wx/Lx^3^]

Feed Conversion Ratio = total feed consumed (g, dry weight)/total weight gain (g, wet weight)

Survival Rate (%) = 100 × [final number of fish/initial number of fish]

where t is the duration of the experiment (days), Lx is the final body length (cm), Wx is the final body weight (g) and Wi is the initial body weight (g).

### 2.7. Blood Biochemistry

Blood samples were centrifuged at 1200 rpm for 10 min to obtain plasma. Aspartate aminotransferase (AST) and alanine aminotransferase (ALT) enzyme activities were determined following the procedure of Bergmeyer et al. [34] and using a kit Liquicolor (Society for Biochemistry and Diagnostics, Wiesbaden, Germany). Samples were incubated 15 min at 37 °C for AST and 5 min at 37 °C for ALT. Absorbance readings were performed with a spectrophotometer for three minutes at an absorbance (ABS) of 340 nm for AST and ALT. Enzyme activity was expressed as UL^−1^.

Plasma glucose, cholesterol and triglycerides [35,36] were assessed with the reagent kit Liquicolor (Society for Biochemistry and Diagnostics, Wiesbaden, Germany) for each variable, respectively. They were then incubated at 37 °C for 25 min in a water bath. The colourimetric reactions changed according to the variable—for cholesterol and triglycerides, it was the tests with lipid lightening factor and for glucose, it was the deproteinisation method. They were then taken to the spectrophotometer to determine absorbance readings at 500 nm. All assays were performed in triplicate to avoid errors as far as possible.

### 2.8. Digestive Enzyme Activity

Lipase, protease and amylase activities were analysed. The specific amylase activity was determined according to the modified method of Bernfeld [37], using starch as substrate and maltose as standard. The extract was evaluated by incubation at 37 °C as follows: 10 µL of extract with 0.25 mL of 1% (*w*/*v*) soluble starch in 0.25 mL of 0.1 M citrate-phosphate buffer with a pH of 7.0. After an incubation time of 30 min, reducing sugars were measured using a UV/visible spectrophotometer with absorbance at 600 nm. Protease activity was performed using the technique described by Walter [38] with 1% casein as substrate in 100 mM of Tris-HCl, 10 mM of CaCl_2_ and a pH of 9.0. The reaction mixture was incubated for 30 min at 37 °C, and the reaction was stopped by adding a 20% TCA solution. The amount of tyrosine (0.005 mL µg^−1^cm^−1^) released in the supernatant was measured at an ABS of 280 nm.

The specific lipase activity was assessed according to the method of Versaw et al. [39], where 100 µL of sodium taurocholate (100 mM) and 1.9 mL (50 mM) of Tris-HCl (pH = 7.2) were added to 20 µL of enzyme extract and incubated at room temperature for 5 min, and the reaction was started with 20 µL of ß-naphthyl caprylate (200 mM) for 30 min at 37 °C. Then, 20 µL of fast blue (100 mM) was added and incubated for 5 min at room temperature. The reaction was stopped with 200 µL of TCA (0.72 N), rinsed with 2.71 mL of ethanol/ethyl acetate (1:1 *v*/*v*) and read in the spectrophotometer with an ABS of 540 nm.

All these digestive enzyme activities were expressed as specific activity (U mg^−1^ of protein). The protein content of the crude intestinal extracts was determined using the method of Bradford [40] with bovine serum albumin (1 mg·mL^−1^) as a standard.

### 2.9. Intestine Histology

Midgut samples were fixed in 10% neutral buffered formalin. After 48 h, tissues were dehydrated in a graded ethanol series (70–100%), covered in paraffin and sectioned at 5 µm on a rotating microtome. They were then stained with haematoxylin and eosin (H&E) and observed under the light of an optical microscope, using a digital colour camera connected to the microscope. Then, on a computer with Image Scion 4.0.2 software, the areas of the intestinal fold tissues were measured.

### 2.10. Gut Microbiota Metagenomics

Samples for isolation of bacterial community DNA were prepared according to previous methods described by Giatsis et al. [41] and Shi et al. [42]. A commercial column-based ZymoBIOMICS DNA kit was used, applying the standard technique for DNA isolation and using PCR amplification to evaluate bacterial 16SrRNA genes. Specific primers were used according to the needs established in the sample record sheet. Primers 341F/805R (5′-CCTACGGGGGNGGCWGCAG-3′ and 5′-GACTACHVGGGTATCTAATCC-3′ accordingly) were used [43,44].

The resulting PCR amplifications were sequenced through the 2 × 300 format of the Illumina MiSeg sequencing platform (Illumina, PE250, San Diego, CA, USA). For phylogenetic analysis of taxonomic classification, the high-throughput algorithm of the Ribosomal Database Project (RDP) classifier described in Wang et al. [45] was employed. The database used was RefSeq RDP 16S V3, based on a set of FASTA files from: https://benjineb.github.io/dada2/training.html (accessed on 18 January 2024) for 16S rRNA gene sequences with DADA2 format for bacteria and archaea (Version 2). The OTU sequence was annotated, and Shannon–Weaver (H) and Simpson (J) diversity indices and Chao’s richness estimates were calculated according to the taxonomic information obtained. Beta diversity was calculated to estimate pairwise sample differences between Tilapia gut microbial communities.

### 2.11. Hypoxia Stress

Six fish/aquaria were used for this test. Each aquarium was considered a replicate. The removal of dissolved oxygen (<0.1 mg·L^−1^) in the water was ensured by adding sodium bisulfite (NaHSO_3_) (0.15 g 500 mL). The fish were exposed to hypoxia for 1 h. Sampling was performed every 5 min, and the number of live and dead organisms was recorded to calculate the relative level of protection (RLP) [46]. Dead fish were removed immediately.

Relative level of protection (%) = [1 − (% mortality of treated fish/% mortality of control fish)] × 100.

### 2.12. Statistical Analysis

The effect of dietary supplementation with organic silicon on growth, feed utilisation, physiological biochemistry, morphohistology, gut microbiota and stress resistance was assessed by analysis of variance (ANOVA) of one-way with a Tukey’s post hoc test using the Minitab^®^ statistical package (Minitab Inc., Philadelphia, PA, USA) at a significance of *p* < 0.05. All results are reported as mean ± standard error (SE).

## 3. Results

### 3.1. Fish Growth Performance

For growth performance and feed utilisation in fish (Table 3), a significant difference (*p* < 0.05) was found for supplementation with organic silicon in the diet; the highest values were found for final weight (29.56 g), final length (13.39 cm), weight gain (22.06 g) and specific growth rate (2.42%) when 50 mg·kg^−1^ of organic silicon is included in the diet. The feed conversion factor (1.27) and condition factor (1.21) were lower with 50 mg·kg^−1^ of organic silicon in the fish’s diets. All treatments with organic silicon and the control showed a survival of 100% (*p* > 0.05).

### 3.2. Blood Biochemistry

With respect to blood biochemistry (Table 4), significant differences (*p* ˂ 0.05) were found in the levels of metabolic enzymes (ALT and AST), cholesterol, glucose and triglycerides between the groups treated with organic silicon and the treatment control. The groups of fish fed organic silicon in the diet showed significantly higher glucose levels (*p* ˂ 0.05) than the control treatment. Blood levels of metabolic enzymes, cholesterol and triglycerides were decreased significantly in all groups of fish treated with diets supplemented with organic silicon (*p* ˂ 0.05) with respect to fish in the control treatment.

### 3.3. Digestive Enzyme Activity

It was found that the activity of digestive enzymes (lipases, proteases and amylases) of fish (Table 5) was significantly influenced by the levels of organic silicon in the diet. Fish fed with levels of 20 to 40 mg·kg^−1^ of organic silicon showed lipase values of 38.62 to 41.39 U mg^−1^ of protein. Protease activity levels (38.94 U mg^−1^ of protein) decreased with 50 mg·kg^−1^. However, for amylases (80.24 U mg^−1^ of protein), the highest values were observed with 50 mg·kg^−1^ of organic silicon in the diet.

### 3.4. Intestinal Morphohistology

**Figure 1:**
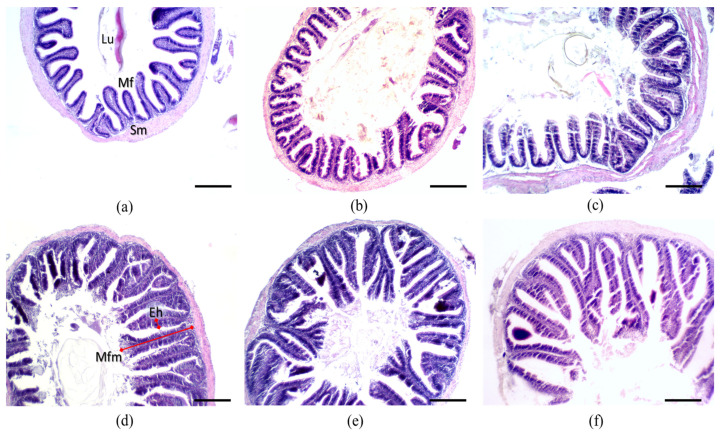
Cross section of the proximal intestine of juvenile hybrid red Tilapia fed diets supplemented with organic silicon. Treatments: (**a**) 0 (control), (**b**) 10, (**c**) 20, (**d**) 30, (**e**) 40 and (**f**) 50 mg·kg^−1^ of organic silicon in the diet. H&E stain. Abbreviations: Lu—Lumen; Sm—subepithelial mucosa; Mf—mucosal fold; Mfm—mucosal fold length; Eh—enterocyte height. The scale bar is 100 µm.

After eight weeks, fish fed various levels of organic silicon showed no characteristics associated with mucosal inflammation and/or intestinal damage. All experimental diets showed evident effects on gut morphometry in juvenile red Tilapia (Figure 1). Fish fed organic silicon showed development and hypertrophy of the apical zone of the mucosal fold. Increased coalescence, few infiltrations of leukocytes and eosinophilic granulocytes were found. Intestinal mucosa was characterised by several epithelial folds where an abundance of cells involved and necessary for digestive processes, such as enterocytes, goblet cells and enteroendocrine cells, were found.

In Table 6 are reported the results of intestinal morphohistology, which shows that for mucosal fold length, there are highly significant differences (*p* < 0.05) with a higher mean (342.56 µm) at 30 mg·kg^−1^ of organic silicon. The subepithelial mucosa and enterocyte height were significantly higher in the 30, 40 and 50 mg·kg^−1^ of organic silicon in the diet, and the number of mucosal folds was significantly higher in the 40 and 50 mg·kg^−1^ of organic silicon in the diet.

### 3.5. Gut Microbiome Metagenomics

Sequencing of the PCR-amplified 16S rRNA gene generated 1,183,683 reads related to the gut-associated microbiota of red Tilapia (*O. mossambicus* × *O. niloticus*) with 81.49% classification of 27 amplicons. The number of operational taxonomic units (OTU) was found to be relatively high for the treatments compared to the control and had a high richness with a number of OTU/Chao-1 of about 533/542 and 384/387 for the treatment (50 mg·kg^−1^) and control (0 mg·kg^−1^) groups, respectively (Table 7). The Shannon–Weaver (H′) and Simpson (1-λ) indices also showed high diversity for all treatments compared to the experimental control.

**Figure 2:**
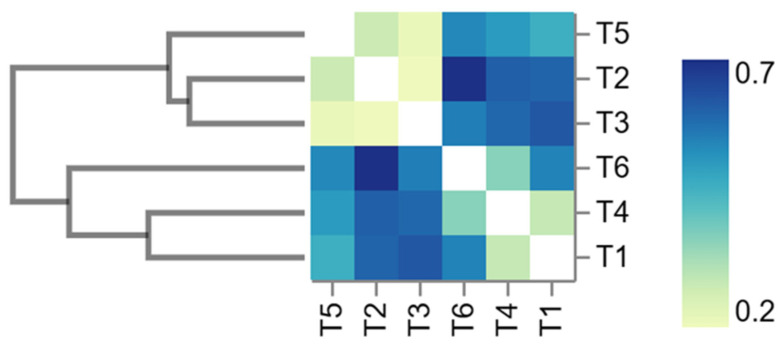
Bray–Curtis cluster tree and diversity distance heat map, showing results of beta diversity analysis at the phylum level of juvenile hybrid red Tilapia fed diets supplemented with organic silicon. Treatments: T1-0, T2-10, T3−20, T4-30, T5-40 and T6-50 mg·kg^−1^ of organic silicon in the diet.

Beta diversity analysis explored differences between samples at each elevation with respect to species complexity. In Bray–Curtis distance values, differences were found in the groups of bacterial communities. They range from 0 (communities with identical structures) to 1 (no similarity). The mean Bray–Curtis distances between samples ranged from 0.28 to 0.74 and showed that beta diversity was highest in the 10, 20 and 40 mg·kg^−1^ treatment samples and lowest in the 0, 30 and 50 mg·kg^−1^ treatment samples. A Bray–Curtis cluster tree and heat map illustrate the beta diversity analysis (Figure 2).

**Figure 3:**
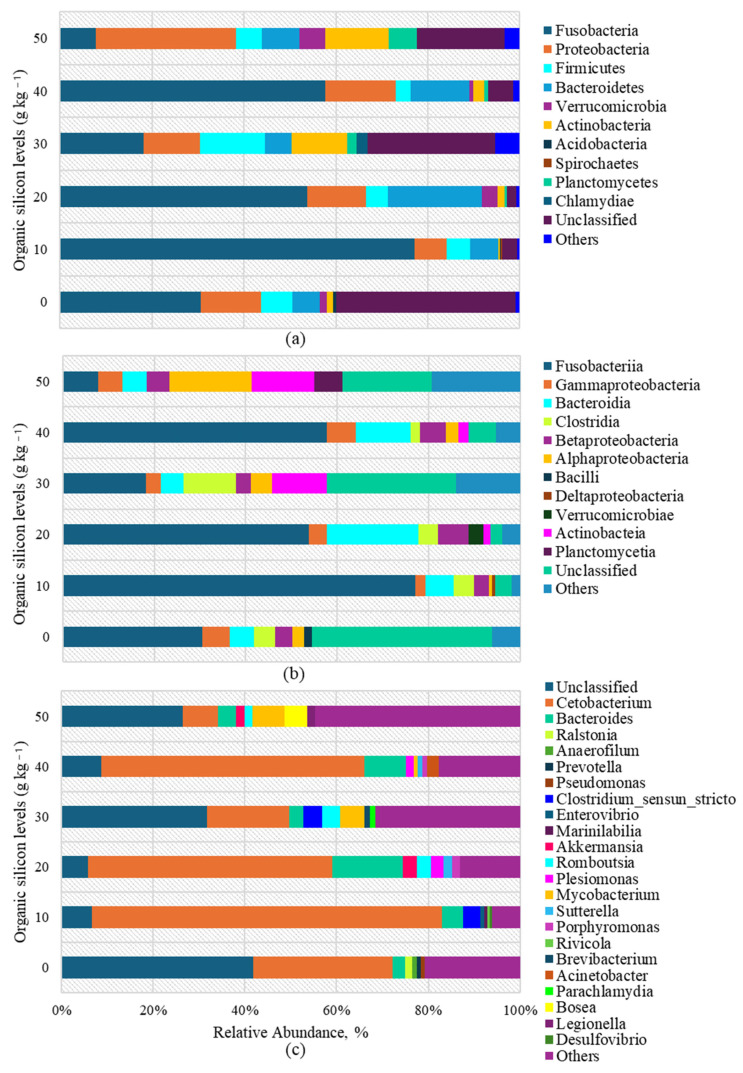
Average relative abundance in dominant bacteria of juvenile hybrid red Tilapia fed diets supplemented with organic silicon. Phylum (**a**), Class (**b**) and Genus (**c**).

The 16S gene-based phylogenetic analysis of intestinal microbiota in the fish from the experimental treatments recorded the presence of 11 phyla, 12 classes and 21 genera. The relative abundance of gut microbiome composition of juvenile red Tilapia was shown to be dominated by the following phyla (Figure 3): Fusobacteria in treatments of 0, 10, 20, 30 and 40 mg·kg^−1^ of organic DOS with values of 30.57%, 77.04%, 53.77%, 18.23% and 57.66%, followed by Proteobacteria in the 50 mg·kg^−1^ treatment, while the rest of the phyla included Planctomycetes, Bacteroidetes, Acidobacteria, Chlamydiae, Verrucomicrobia, Spirochaetes, Firmicutes and Actinobacteria.

Relative abundance in terms of classes was found to be dominated in 0, 10, 20, 30 and 40 mg·kg^−1^ of DOS by Fusobacteria (30.57%, 77.04%, 53.77%, 18.23% and 57.66%), while at 50 mg·kg^−1^ of DOS, Gammaproteobacteria predominate (24.52%) according to Figure 2. The remaining classes included Costridia, Bacilli, Actinobacteia, Betaproteobacteria, Deltaproteobacteria, Planctomycetia, Bacteroidia, Alphaproteobacteria and Verrucomicrobiae.

The relative abundance of the genus showed to be dominated by Cetobacterium, with no significant difference (*p* > 0.05) for this genus between 10, 20 and 40 mg·kg^−1^ of DOS (74.41%, 53.34% and 57.29%) but not with respect to 0, 30 and 50 mg·kg^−1^ of DOS (30.34%, 18.04% and 7.70%), respectively. The remaining genera comprise Anaerophilum, Enterovibrio, Mycobacterium, Acinetobacter, Prevotella, Akkermansia, Sutterell, Parachlamydia, Bacteroides, Pseudomonas, Romboutsia, Porphyromonas, Bosea, Ralstonia, Clostridium, Plesiomonas, Brevibacterium and Legionella.

### 3.6. Stress Resistance

The mortality rate of juvenile red Tilapia, after challenge, was also significantly affected by the dietary treatments with organic silicon. During a 60 min observation period, mortality was 77.78% in the control group, while the five groups treated with organic silicon had mortality rates of 38.89% to 16.67%, respectively. The relative protection level (RPL) in the groups treated with organic silicon at 50 mg·kg^−1^ was 78.57% with a significant difference (*p* < 0.05) compared to the other treatments, as shown in Table 8.

## 4. Discussion

Tilapia aquaculture faces challenges for improving zootechnical and health performance. Despite the efforts in tilapia to surpass this issue, the use of organic silica as a dietary additive has been scarcely investigated. The results of this study highlight the potential dietary use of organic silica in farmed tilapia and provide scientific evidence on its safety utilisation and effects on productive performance, blood biochemistry, digestive enzymology, gut microbiota and stress resistance in *O. mossambicus* × *O. niloticus*.

### 4.1. Fish Growth Performance

Organic silica has no toxic elements or lethal anti-nutritional factors [17,47], considering that survival was 100% in the investigated treatments at eight weeks of tilapia culture. It was demonstrated that the progressive inclusion of up to 50 mg·kg^−1^ of DOS significantly improved the growth indicators, feed utilisation and survival of hybrid tilapia (*O. mossambicus* × *O. niloticus*). Treatments with 40 and 50 mg·kg^−1^ of organic silica inclusion in tilapia diets were found to have the highest values for weight and final gain, and the lowest values for FCF (<1.32), which translates into lower feed consumption and higher fish growth. Our results are very similar to those obtained in other investigations carried out under controlled conditions in juvenile red tilapia [48,49]. However, the effect of silicon on fish may vary depending on species, age and aquatic environment [20]. In this regard, silicon has been found to enhance leptin response on the activity of the digestive system related to the regulation of food intake and energy balance, which can affect growth performance [23]. In addition, silicon effect on fish growth could be related to the health of the mucous membranes of the gastrointestinal tract, by stimulating goblet cells for the formation of mucus and Paneth cells for the secretion of digestive enzymes, which improves the digestion and absorption of nutrients [17,24]. These effects on growth and bioindicators coincided with the dietary administration of copper, iron oxide and zinc oxide nanoparticles in tilapia (*O. niloticus*) [50,51]. Moreover, silica nanoparticles in diets improved the growth of tilapia, indicating that the effects of silica at macro and nano-scale on fish growth could be similar [20,52].

The above studies coincide with our results, where fish fed with organic silicon showed better growth performance and feed conversion factor, decreasing the loss of nutrients from the feed. The improved growth performance in our study could be associated with the property of silica to bind with several nutrients to facilitate absorption across the enterocytes and release into the bloodstream to support metabolic processes involved with muscle hyperplasia and hypertrophia [19]. Therefore, the results of our study demonstrated that dietary organic silicon (DOS) may serve as a growth promoter additive for tilapia aquaculture.

### 4.2. Blood Biochemistry

Blood physiological indicators help to know the health status of fish supplemented with a given additive. In our study, metabolic enzymes such as AST and ALT in fish consuming DOS showed lower values than the control. These blood enzymes are indicators of liver function and markers of hepatotoxicity [53], which means that fish fed silicon, even at the highest doses, did not show liver damage. In comparison, in rats supplemented with silica nanoparticles, ALT and AST levels increased significantly and correlated with inflammatory cell infiltration and adipose tissue degeneration in the liver, especially in the 500 mg·kg^−1^ dose group [54]. In our work, the doses were lower (40 and 50 mg·kg^−1^ of organic silicon), and the results coincided with those reported by Alandiyjany et al. [52] and Mahboub et al. [28] in *Clarias gariepinus* and *O. niloticus*, demonstrating that silicon in adequate doses is a safe, favoured metabolic activity and can mitigate hepatic dysfunction. On the other hand, glucose decreased up to the 30 g·kg^−1^ organic silicon treatment and then increased in the 40 and 50 g·kg^−1^ treatments relative to the control. The reduction of plasma glucose in fish may suggest an increase in metabolic rate [55,56].

However, all levels found are within the normal values for red tilapia [57,58] and suggest that the glucose balance was appropriate for tilapia. In contrast, triglycerides and cholesterol levels decreased in fish supplemented DOS with respect to the control fish. Therefore, organic silicon had a hypocholesterolemic effect [59], probably through the regulation of fat digestion, absorption and metabolism in tilapia. In line with these findings, dietary silicon in rats reduced cholesterolaemia and the intestinal absorption of cholesterol and triglycerides by reducing acetyl-coenzyme A acetyltransferase-2 (ACAT2) activity and affecting bile acid production, provoking an increase in cholesterol excretion [60,61]. Based on the results obtained in our work, silicon may have contributed in the intestine to the process of micelle solubilisation. This leads to reduced cholesterol esterification in enterocytes by inhibiting the enzyme acyl-CoA:cholesterol acyltransferase (ACAT), which prevents cholesterol and triglycerides absorbed by enterocytes from being immediately incorporated into chylomicrons, allowing unesterified cholesterol to be sent back to the intestine.

### 4.3. Digestive Enzyme Activity

Some studies [62,63] have indicated that the use of additives in diets can exert biostimulatory effects on neuropeptides, such as secretin and cholecystokinin, which directly influence the synthesis and secretion of bile acids and zymogen-rich pancreatic juices necessary for feed hydrolysis. The expression of digestive enzymes is directly related to the digestion and absorption of nutritional material and animal growth [12]. In our study, a significant increase in digestive enzyme activity (proteases, lipases and amylases) was found in silicon treatments compared to the control, which may be attributed in part to more active neuropeptide receptors and increased stimulation of pancreatic acini responsible for the secretion of digestive zymogens for the hydrolysis of molecules (proteins, lipids and carbohydrates) that come from the feed. These findings are in line with the results reported by Hassaan et al. [64,65], who used dietary mineral additives on *O. niloticus* and found significant increases in digestive enzyme activities.

On the other hand, silicon possesses antibacterial activity that may decrease the incidence of pathogens [66], favouring intestinal health by stabilising the intestinal barrier and inducing the expression of intestinal digestive enzymes from the brush border of enterocytes, thus increasing the hydrolysis of molecules from feed and the absorption by intestinal microvilli [64,67].

### 4.4. Intestinal Morphohistology

The histological results in our work support the biochemical results, showing that organic silicon improves digestion and physiology in tilapia without causing tissue damage. The general morphology of the intestine in all groups investigated was similar and within the normal range for the species. Intestinal morphohistological analysis is a reliable method to determine the impact of diet and additives on the absorptive capacity of the intestine in fish, as well as intestinal immunity [68,69].

Increased subepithelial mucosa, intestinal folds, enterocyte height and muscle layer thickness in our work were positively influenced in the fish treated with dietary silicon, which is a good indicator of a healthy gut. In agreement with our findings, increased gut fold number and size improved digestibility, absorption and utilisation of nutrients in the fish gut [70]. Moreover, mucosal barrier integrity is crucial for maintaining healthy tissue homeostasis. For instance, among the specialised cells in the fish intestine, microfold cells play a key function in the uptake of food-derived antigens and pathogens. Mucus secreted by goblet cells provides the first layer of intestinal protection to keep the integrity of the mucosal barrier, which also depends on cell proliferation to replace damaged cells [71]. In our work, subepithelial mucosa, enterocyte height, mucosal fold length and the number of mucosal folds were improved in fish fed organic silicon. In contrast, Bashar et al. [20] reported that feeding silicon nanoparticles to *Oreochrimis niloticus* fish did not enlarge the folds; however, it did widen them, thus significantly increasing the surface area for absorption, which may be related to the dosage of silicon included in the diet. The results in our work and [20] indicate a better surface area for nutrient enzymatic digestion and absorption in tilapia supplemented with organic silicon. Those morphological changes can be associated with cell turnover or a concomitant increase in cell migration and an improved function of enterocytes (absorptive cells) since they act as a primary structural component of the intestinal epithelium and regulate the molecular digestion and passage of nutrients [69,72,73].

### 4.5. Gut Microbiome Metagenomics

In our study, after fish were fed DOS for eight weeks, the diversity and composition of the gut microbiota increased. The gut microbiota is closely associated with host health [74], but this aspect has also been one of the less explored in aquaculture. The phyla Fusobacteria, Proteobacteria and Bacteroidetes are among the main groups that dominate the gut microbiota of cichlids. They can represent up to 90% of the communities and be part of both allochthonous (transient) and autochthonous (adherent) microbiota [75,76], as corroborated by the results obtained in our work. According to Kamble et al. [77], Fusobacteria are bacilli that act as commensals and constitute one of the main groups of the microbiota of the digestive tract. They produce enzymes, such as proteases, peptidases and hydrolases, allowing them to use proteins as an energy source, which subjects amino acids to deamination and decarboxylation. To this phylum belongs the important genus Cetobacterium, which was the most abundant in the intestinal microbiota of our study.

Together with other microorganisms, they are essential for the digestive process. It has been reported to participate in the production of vitamin K, the normal function of the central nervous system, the formation of erythrocytes in the blood and the synthesis of several proteins [78,79,80]. Fusobacteria can ferment carbohydrates, amino acids and peptides, producing various organic acids, such as acetic, propionic, butyric, formic and succinic acids, depending on the species of bacteria and the substrate [81]. Our study shows that the highest relative abundance of Fusobacteria was in the groups with 10, 20 and 40 mg·kg^−1^, which was consistent with the best histological performance in the fish. Another phylogenetic group was Bacteroidetes, which promotes energy metabolism, ferments undigestible polysaccharides and carbohydrates and produces short-chain fatty acids [82,83]. Within this phylum and class, the genus Bacteroides was found in the highest abundance. Bacteroides species have been reported to be important producers of B12 in the gut of animals [74,80], underlining the importance of maintaining a balanced microbiome for healthy tilapia. With regard to Proteobacteria, they are involved in carbon complexes and nitrogen degradation [82] and also have an inhibitory effect against bacterial and fungal pathogens [84].

Zhang et al. [85] demonstrated in rainbow trout (*Oncorhynchus mykiss*) that infection by Ichthyophthirius multifiliis occurred after microbial dysbiosis, which was mainly characterised by a decrease in Proteobacteria in the skin. Several opportunistic pathogens exist in the tilapia gut, such as Aeromonas and Vibrio. In this study, only in the control treatment were Pseudomonas and Enterovibrio found; the latter was also found in the treatment with 10 mg·kg^−1^ of DOS but not in the remaining treatments. Thus, organic silicon in the remaining doses could reduce the abundance of pathogens in the intestines of the fish. Wang et al. [86] in their study found that dietary minerals can act as prebiotics, contributing to the balance and strengthening of tilapia gut microbiota. In our study, the results of the gut microbiota analysis correlated with the histological results, which may be attributed to the fact that silicon was able to cholinergically stimulate enteroendocrine cells and autonomic secretion in goblet cells, which are responsible for secreting mucus. Mucus coats the intestinal mucosa, where bacterial communities and Paneth cells secrete lysozyme and defensive antimicrobial peptides, such as α and β, and contribute to good intestinal health and bacterial population, respectively. In this regard, it has been reported that the number of Paneth cells and the amount of mucus secretion increase as a consequence of an increased bacterial load [87]. Therefore, the study of gut microbial communities by deep sequencing helps to explain the modulating effect of organic silicon in the tilapia gut; and the results in this study suggest that organic silicon may promote tilapia growth and health by regulating the abundance of gut microbiota.

### 4.6. Hypoxia Stress

It is also important to consider hypoxia extremes, as low levels and/or loss of oxygen in aquaculture culture systems are critical problems, especially in intensive systems. A complex process of physiological and biochemical changes is involved in fish to cope with hypoxia stress [88]. In our study, fish subjected to extreme hypoxia swam to the surface in search of oxygen. We also found that tilapia swimming slowed down under hypoxia stress, which is similar to reports on Atlantic cod (*Gadus morhua*) [89]. This appears to be an adaptive behaviour that fish display to reduce energy and oxygen consumption under hypoxia stress. Fish under acute hypoxia stress generally reduce oxygen consumption by slowing down their movements and improving oxygen-carrying capacity by increasing red blood cell and haemoglobin concentration [90]. In this regard, fish supplemented with DOS treatments may have produced a greater response than the control diet, as previously reported in *O. niloticus* where erythrocyte and haemoglobin levels increased in fish fed silicon [20].

In both vertebrates and invertebrates, glycogen metabolism regulation is associated with environmental stress and is the main pathway for energy acquisition [91]. In our work, although the fish’s blood glucose levels prior to hypoxia exposure were within the ranges for the species, those treatments with higher dietary silicon levels had higher blood glucose levels, which correlated with survival and RPL scores upon hypoxia exposure. It has been documented that hypoxia activates anaerobic metabolism and that anaerobic glycolysis would cover the high energy requirement of animals during hypoxia stress [92]. Due to the low ATP yield of anaerobic glycolysis, substrates, such as glycogen and glucose, are substantially consumed [14], which may have occurred in our work. That is, hypoxia stress would induce fish to swim towards the water’s surface, slow down their movements and change their behavioural patterns.

## 5. Conclusions

This study revealed that dietary supplementation with organic silicon on the growth performance, blood biochemistry, digestive enzymes, morphohistology and intestinal microbiota and stress resistance in hybrid Tilapia (*O. mossambicus* × *O. niloticus*) had no negative effects or severe changes on the physiology, metabolism or gut morphology of the fish. The results showed that up to 50 mg·kg^−1^ of organic silicon can be applied to the diet of red Tilapia without adverse health effects. The interactions between growth performance, physiological digestive and metabolic capacity in hybrid juvenile red Tilapia still deserve further exploration. Considering that this varies with each species, stage of development, culture conditions and diet, each case must be investigated separately. A study that considers the use of the best diet with larger specimens or specimens under different physiological conditions should be performed to obtain a better understanding of the levels of supplementation with organic silicon in the diet.

## Figures and Tables

**Table 1:** Formulations of experimental diets with the inclusion of organic silicon at different levels.

Ingredients (g·kg^−1^)	Organic Silicon Levels (mg·kg^−1^)						Cost (USD·kg^−1^)
	0	10	20	30	40	50	
Fish meal ^1^	245	245	245	245	245	245	1.91
Soybean meal ^2^	260	260	260	260	260	260	0.64
Wheat flour ^3^	210	210	210	210	210	210	0.50
Corn meal ^4^	209.8	208.8	207.8	206.8	205.8	204.8	0.44
Organic silicon ^5^ (mg·kg^−1^)	0	10	20	30	40	50	22.50
Vegetable oil ^6^	10	10	10	10	10	10	0.80
Fish oil ^7^	15	15	15	15	15	15	1.20
Gelatine ^8^	19	19	19	19	19	19	0.50
Mineral premixes ^9,10^	10	10	10	10	10	10	4.75
Vitamin premixes ^11,12^	20	20	20	20	20	20	4.75
Ascorbic acid	1	1	1	1	1	1	21.64
Antioxidant BHT ^13^	0.2	0.2	0.2	0.2	0.2	0.2	13.50

The ingredients were bought from: ^1^ Comercial “El Gordillo” in Santo Domingo de los Tsáchilas, Ecuador; ^2^ Ullón Poultry in Valencia, Ecuador; ^3,4,6^ Supermaxi in Quevedo, Ecuador, ^5^ Organika in Santo Domingo de los Tsáchilas, Ecuador; ^7^ Fortidex S.A. in Santa Elena, Ecuador; ^8,9,11,13^ Super Éxito in Quevedo, Ecuador; ^10^ This premix contained the following in mg·kg^−1^: magnesium sulfate 5.1; sodium chloride 2.4; potassium chloride 2; ferrous sulfate 1; zinc sulfate 0.2; cupric sulfate 0.0314; manganous sulfate 0.1015; cobalt sulfate 0.0191; calcium iodate 0.0118; and chlorine chloride 0.051. ^12^ This premix contained the following in mg·kg^−1^: thiamine 60; riboflavin 25; niacin 40; vitamin B6 50; pantothenic acid 75; biotin 1; folate 10; vitamin B12 0.2; Vitamin K5 0.1; Hill 600; myoinositol 400; vitamin C 200; vitamin A 5000 IU; vitamin E 100; and vitamin D 0.1.

**Table 2:** Chemical composition and essential amino acids of diets with the inclusion of organic silicon.

Real Proximal Composition (g·kg^−1^, as Fed Basis) ^1^	Organic Silicon Levels (mg·kg^−1^)					
	0	10	20	30	40	50
Dry matter (DM)	925.0	927.5	936.5	929.5	929.1	929.0
Crude protein	323.8	323.0	322.2	311.6	320.1	319.5
Crude lipid (CL)	57.5	58.2	57.9	57.5	57.1	56.8
Crude fibre	15.1	17.4	18.3	20.4	25.1	27.3
Ash	102.9	103.6	106.1	108.4	109.3	111.5
Nitrogen-free extract (NFE)	425.7	425.3	432.0	431.6	417.5	413.9
Digestible energy (MJ kg^−1^ food)	12.29	12.28	12.29	12.29	12.3	12.30
PC ED^−1^ (mg PC MJ^−1^)	26.35	26.29	26.22	25.35	26.03	25.97
Calculated essential amino acids (%, as fed basis)						
Threonine	1.45	1.45	1.44	1.43	1.42	1.42
Valine	1.63	1.63	1.61	1.59	1.58	1.56
Methionine	0.73	0.73	0.73	0.73	0.73	0.73
Isoleucine	1.32	1.32	1.32	1.31	1.31	1.30
Leucine	2.37	2.37	2.36	2.35	2.34	2.33
Lysine	1.85	1.85	1.83	1.81	1.80	1.79
Histidine	0.85	0.85	0.84	0.84	0.84	0.84
Arginine	1.40	1.40	1.39	1.39	1.38	1.38
Tryptophan	0.33	0.33	0.33	0.32	0.32	0.32
Phenylalanine	1.21	1.21	1.20	1.19	1.18	1.18

^1^ Data are reported as means of three replicates.

**Table 3:** Production rates of juvenile hybrid red Tilapia fed diets supplemented with organic silicon.

Productive Parameters	Organic Silicon Levels (mg·kg^−1^)						*p*-Value
	0	10	20	30	40	50	
IW (g)	7.50 ± 3.35 ^a^	7.51 ± 3.53 ^a^	7.49 ± 3.14 ^a^	7.49 ± 3.09 ^a^	7.51 ± 3.47 ^a^	7.50 ± 3.79 ^a^	0.9999
FW (g)	25.05 ± 5.05 ^b^	25.57 ± 6.36 ^b^	26.38 ± 5.85 ^ab^	25.46 ± 6.79 ^b^	28.71 ± 6.94 ^ab^	29.56 ± 7.30 ^a^	0.0026
FL (cm)	11.94 ± 1.00 ^b^	11.93 ± 1.24 ^b^	12.25 ± 1.22 ^b^	11.93 ± 1.32 ^b^	12.19 ± 1.33 ^b^	13.39 ± 1.28 ^a^	0.0001
WG (g)	17.55 ± 6.95 ^b^	18.06 ± 0.89 ^b^	18.88 ± 0.12 ^ab^	17.97 ± 0.28 ^b^	21.20 ± 0.51 ^a^	22.06 ± 0.96 ^a^	0.0430
SGR	2.15 ± 0.13 ^c^	2.19 ± 0.17 ^c^	2.26 ± 0.17 ^b^	2.19 ± 0.09 ^c^	2.35 ± 0.19 ^a^	2.42 ± 0.10 ^a^	0.0335
CF	1.47 ± 0.08 ^b^	1.51 ± 0.03 ^b^	1.43 ± 0.04 ^b^	1.50 ± 0.05 ^b^	1.53 ± 0.02 ^b^	1.21 ± 0.02 ^a^	0.0001
FCF	1.48 ± 0.09 ^a^	1.44 ± 0.01 ^ab^	1.41 ± 0.07 ^abc^	1.46 ± 0.03 ^ab^	1.32 ± 0.03 ^bc^	1.27 ± 0.04 ^c^	0.0030
SR (%)	100 ± 0.00 ^a^	100 ± 0.00 ^a^	100 ± 0.00 ^a^	100 ± 0.00 ^a^	100 ± 0.00 ^a^	100 ± 0.00 ^a^	0.4910

Results are reported as mean ± SE of three groups by treatment (*n* = 3). ^abc^ Different letters in the superscripts of the means of the same row denote significant differences (*p* < 0.05). Abbreviations: IW—Initial Weight; FW—Final Weight; FL—Final length; WG—Weight Gain; SGR—Specific growth rate; FCF—Feed conversion factor; CF—Condition Factor; SR—Survival Rate.

**Table 4:** Metabolic biochemistry in blood of juvenile hybrid red Tilapia fed diets supplemented with organic silicon.

Blood Biochemistry	Organic Silicon Levels (mg·kg^−1^)						*p*-Value
	0	10	20	30	40	50	
ALP (UL^−1^)	41.50 ± 1.50 ^ab^	39.50 ± 0.05 ^b^	38.00 ± 3.00 ^b^	41.67 ± 0.57 ^ab^	45.00 ± 3.26 ^a^	42.50 ± 2.04 ^ab^	0.0460
AST (UL^−1^)	140.45 ± 4.25 ^a^	96.32 ± 3.22 ^b^	92.46 ± 2.46 ^bc^	107.30 ± 5.21 ^b^	86.45 ± 1.75 ^c^	89.94 ± 2.43 ^c^	0.0064
Glucose (mg dL^−1^)	73.00 ± 3.00 ^b^	67.33 ± 2.52 ^bc^	62.00 ± 4.00 ^c^	63.00 ± 4.00 ^c^	77.33 ± 0.58 ^a^	79.00 ± 1.00 ^a^	0.0001
Cholesterol (mg dL^−1^)	79.33 ± 4.51 ^a^	77.33 ± 2.52 ^a^	67.33 ± 1.53 ^b^	66.33 ± 1.53 ^b^	67.33 ± 2.52 ^b^	69.00 ± 5.57 ^b^	0.0186
Triglycerides (mg dL^−1^)	204.00 ± 4.00 ^a^	190.00 ± 5.00 ^b^	187.00 ± 3.00 ^b^	195.33 ± 0.58 ^ab^	193.33 ± 3.51 ^ab^	192.33 ± 5.86 ^b^	0.0044

Results are reported as mean ± SE of three groups by treatment (*n* = 3). ^abc^ Different letters in the superscripts of the means of the same row denote significant differences (*p* < 0.05). Abbreviations: ALT—alanine aminotransferase; AST—aspartate aminotransferase.

**Table 5:** Digestive enzyme activity of juvenile hybrid red Tilapia fed diets supplemented with organic silicon.

Enzyme Activity (U mg^−1^ of Protein)	Organic Silicon Levels (mg·kg^−1^)						*p*-Value
	0	10	20	30	40	50	
Proteases	37.84 ± 1.80 ^a^	42.09 ± 2.80 ^a^	51.96 ± 3.10 ^b^	54.03 ± 2.70 ^b^	52.91 ± 2.60 ^b^	38.94 ± 2.20 ^a^	0.0001
Lipases	35.92 ± 2.70 ^ab^	34.01 ± 3.70 ^a^	39.62 ± 1.50 ^b^	41.39 ± 1.60 ^b^	38.84 ± 1.20 ^b^	38.62 ± 1.00 ^b^	0.0049
Amylases	71.00 ± 2.20 ^ab^	71.72 ± 2.20 ^ab^	66.89 ± 3.00 ^a^	68.34 ± 1.40 ^a^	75.45 ± 1.90 ^bc^	80.24 ± 2.00 ^c^	0.0002

Results are reported as mean ± SE of three groups by treatment (*n* = 3). ^abc^ Different letters in the superscripts of the means of the same row denote significant differences (*p* < 0.05).

**Table 6:** Intestinal morphohistology of juvenile hybrid red Tilapia fed diets supplemented with organic silicon.

Intestinal Morphohistology	Organic Silicon Levels (mg·kg^−1^)						*p*-Value
	0	10	20	30	40	50	
SM (µm)	16.89 ± 1.35 ^cd^	16.30 ± 0.06 ^d^	17.47 ± 0.89 ^bcd^	19.85 ± 0.49 ^ab^	19.45 ± 1.00 ^abc^	21.09 ± 1.64 ^a^	0.0006
EH (µm)	14.75 ± 1.50 ^b^	16.20 ± 0.05 ^ab^	17.75 ± 0.50 ^ab^	19.59 ± 0.37 ^a^	17.44 ± 2.10 ^ab^	20.09 ± 2.30 ^a^	0.0055
MFM (µm)	259.37 ± 2.00 ^e^	283.17 ± 2.14 ^d^	294.08 ± 2.68 ^c^	342.56 ± 2.41 ^a^	309.00 ± 0.08 ^b^	304.57 ± 1.5 ^b^	0.0001
NMF	37.36 ± 1.00 ^bc^	32.43 ± 0.64 ^d^	39.61 ± 1.07 ^bc^	35.32 ± 2.88 ^cd^	40.41 ± 1.00 ^ab^	44.52 ± 2.44 ^a^	0.0001

Results are reported as mean ± SE of three groups by treatment (*n* = 3). ^abcd^ Different letters in the superscripts of the means of the same row denote significant differences (*p* < 0.05). Abbreviations: SM—subepithelial mucosa; EH—enterocyte height; MFM—mucosal fold length; NMF—number of mucosal folds.

**Table 7:** Alpha diversity in the gut microbiota of juvenile hybrid red Tilapia fed diets supplemented with organic silicon.

Alpha Diversity	Organic Silicon Levels (mg·kg^−1^)						*p*-Value
	0	10	20	30	40	50	
OTUs	384 ± 14.34 ^c^	387 ± 19.62 ^c^	433 ± 13.63 ^b^	457 ± 21.51 ^b^	403 ± 10.30 ^b^	533 ± 15.29 ^a^	0.037
Shannon (H′)	1.86 ± 0.34 ^b^	1.01 ± 0.48 ^c^	1.77 ± 0.23 ^b^	2.46 ± 0.28 ^a^	1.84 ± 0.36 ^b^	2.84 ± 0.51 ^a^	0.000
Simpson (1-λ)	0.07 ± 0.008 ^b^	0.06 ± 0.005 ^b^	0.07 ± 0.033 ^b^	0.09 ± 0.005 ^a^	0.08 ± 0.028 ^ab^	0.09 ± 0.009 ^a^	0.002
Chao-1	397 ± 11.2 ^c^	405 ± 10.57 ^c^	468 ± 18.43 ^b^	482 ± 13.51 ^b^	489 ± 8.97 ^b^	542 ± 17.52 ^a^	0.007
RMS	110,335	269,430	294,902	147,553	187,822	172,741	

Results are reported as mean ± SE of three groups by treatment (*n* = 3). ^abc^ Different letters in the superscripts of the means of the same row denote significant differences (*p* < 0.05). Abbreviations: OTUs—operational taxonomic units, RMS—readings of means in sequences.

**Table 8:** Hypoxia stress resistance of juvenile hybrid red Tilapia fed diets supplemented with organic silicon.

Hypoxia Stress Response (%)	Organic Silicon Levels (mg·kg^−1^)						*p*-Value
	0	10	20	30	40	50	
Mortality rate	77.78 ^d^	38.89 ^c^	33.33 ^bc^	38.89 ^c^	27.78 ^ab^	16.67 ^a^	0.000
RPL	-	50.00 ^c^	57.14 ^bc^	50.00 ^c^	64.29 ^ab^	78.57 ^a^	0.031

Results are reported as mean ± SE of three groups by treatment (*n* = 3). ^abcd^ Different letters in the superscripts of the means of the same row denote significant differences (*p* < 0.05). Abbreviations: RPL—Relative protection level.

## Data Availability

Data are contained within the article. The supportive data of the findings of this study are available from the corresponding author upon request.
